# Water–Rock Interaction and Freeze–Thaw Cycles as Drivers of Acid Rock Drainage Generation by a Rock Glacier in the European Alps

**DOI:** 10.1021/acsestwater.4c00263

**Published:** 2024-11-13

**Authors:** Boris Ilyashuk, Elena Ilyashuk

**Affiliations:** Department of Ecology, University of Innsbruck, Technikerstrasse 25, 6020 Innsbruck, Austria

**Keywords:** mountain permafrost, groundwater aquifers, ice segregation, rock disintegration, accessory minerals, chemical weathering, trace metals

## Abstract

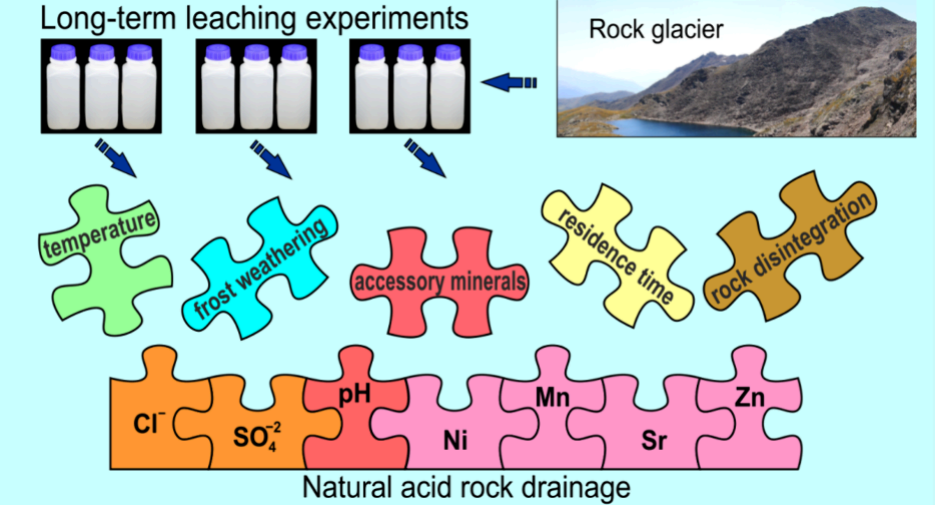

Little is known to date about the processes governing natural acid rock drainage (NARD) generated by rock glaciers. We used paragneiss samples from a catchment with NARD generated by a rock glacier in the Italian Alps for long-term leaching experiments under conditions that are possible within rock glaciers. The findings clearly suggest that at a low acid neutralization capacity of the rock, the dissolution of sulfide minerals, even if they are present in trace amounts, may be the most important process that controls the groundwater acidity at 1 °C, a typical temperature of groundwater discharge from rock glaciers. The acidic conditions increase the solubility and mobility of aquifer lithology-specific trace elements, and concentrations of some heavy metals of geogenic origin (e.g., Mn and Ni) may greatly exceed health standards after a six month interaction of water with paragneisses. Diurnal freeze–thaw cycles were found to be 6–7 times more effective in transformation of coarse rock fragments to fine-grained debris with fresh, reactive mineral surfaces, compared with temperatures above freezing. Cyclic freezing favors an enhanced formation of amorphous silica, a highly effective adsorbent for metal ions, and its redissolution within unfrozen layers of rock glaciers may represent an additional source of trace elements.

## Abstract 2

This study revealed the critical role of prolonged water−rock interaction and cyclic freezing conditions in generation of natural acid rock drainage within a rock glacier-dominated catchment.

## 1 Introduction

Significant impacts of climate change on mountain regions are already occurring at the current level of global warming. Components of the mountain cryosphere (glaciers, permafrost, seasonally frozen ground, etc.) are particularly sensitive and vulnerable to climate change.^1^ With global warming, mountain glaciers around the world are receding, shifting the predominant dynamics of the mountain cryosphere from glacial to periglacial.^2^ Since the 1980s, the extensive and accelerating degradation of thawing permafrost has been increasingly reported for high-elevation areas.^1,3^

Permafrost degradation increases hydraulic conductivity of surface and subsurface materials and therefore accentuates the role of local groundwater flow systems in headwater catchments.^4,5^ One of the most profound consequences of permafrost thaw is a shift from predominately shallow, warm-season flow processes toward deeper year-round flow processes, or in other words, a transition from surface water dominated systems to groundwater-dominated hydrological systems.^4−7^ The shifts in hydrology propagate to changes in the geochemical processes. Increases in groundwater contribution result in greater dissolved load, compared with surface waters, due to longer water–rock contact time at mineral weathering.^8,9^

The most common types of groundwater aquifers in high-elevation areas are represented by typical alpine landforms consisting generally of coarse sediments such as talus, moraine, and rock glacier.^10^ Despite the high hydraulic conductivity of coarse surficial sediments, these shallow aquifers are characterized by the presence of low-conductivity zones within or under coarse sediments in the form of fine sediments or fractured bedrock, where groundwater tends to have a long residence time.^5,10,11^ The chemical composition of groundwaters is determined by the interaction of the groundwater with the aquifer-specific mineralogy along the flow path. Particularly important is the presence, even in trace amounts, of carbonate, sulfate, and sulfide minerals because their dissolution rates are typically orders of magnitude faster than for silicate minerals.^12,13^ And although these minerals are usually present at low abundance in most rocks, even a small proportion of them can dominate solute production.^13−15^

At a relatively low acid-neutralizing potential of rocks, especially in the absence or low abundance of carbonate minerals, the dissolution of pyrite (FeS_2_), pyrrhotite (Fe_1–*x*_S, *x* = 0 to 0.12), and some other sulfide minerals can generate natural acid rock drainage (NARD), a natural process resulting in the formation of acid-sulfate waters with toxic concentrations of trace metals.^16,17^ These minerals are unstable in the presence of oxygen and water and tend to oxidize in relatively oxygen-rich groundwater that circulates through shallow aquifers, forming sulfuric acid. At the same time, the low pH of water facilitates increased mobility of metals^18^ commonly incorporated into the crystal structures of pyrite and pyrrhotite in minor to trace amounts (e.g., Co, Ni, As, Zn, and Cu).^19^ Concentrations of some mobilized metals associated with NARD are often anomalously high and greatly exceed the health standards.

Rock glaciers, shallow groundwater aquifers with potentially high storage capacity and residence times of several months to two years,^11,20^ can be important local sources of changes in water chemistry.^21−23^ Their coarse-grained sediments and upper coarse-blocky openwork structure provide access of atmospheric O_2_ and O_2_-rich water for oxidation of sulfide minerals and generate complex internal thermal regimes within interclast voids.^22^ Over the past decade, there is increasing evidence of NARDs generated by intact rock glaciers (i.e., with permafrost ground ice) within crystalline metamorphic rocks mainly composed of silicate minerals, where sulfides are present in only small amounts, as accessory minerals. Examples of such NARDs are described from headwater areas around the world.^24−29^ Crystalline rocks represent about one-third of the Earth’s surface.^30^ In view of this and taking into account ongoing climate changes and the ubiquitous nature of rock glaciers in high mountain systems,^31^ NARDs generated by rock glaciers may potentially become a serious environmental challenge in the future.

The generation of NARD in nature depends on many different factors and it is very difficult to disentangle the effect of each individual factor,^32^ especially in complex aquifer systems such as rock glaciers. Although the issue of NARDs generated by rock glaciers is not new for the scientific community, there still exists a substantial knowledge gap regarding the factors governing this phenomenon.^21,22,29^ It takes considerable time to accumulate the necessary data to bridge this gap. The aim of this research was to explore the mechanisms of acid generation and trace element mobilization resulting from long-term interaction of water with gneiss, one of the most widely distributed crystalline metamorphic rocks that often constitute shallow bedrock aquifers like rock glaciers in the European Alps. We used paragneiss samples from a headwater catchment with NARD generated by a rock glacier in the Central Eastern Alps^24,25^ for parallel long-term leaching experiments under controlled laboratory conditions: constant liquid-to-solid (L/S) ratio, fixed contact time and temperatures, and the presence or absence of diurnal freeze–thaw cycles (FTCs).

## 2 Materials and Methods

### 2.1 Site Description and Rock Sampling

**Figure 1:**
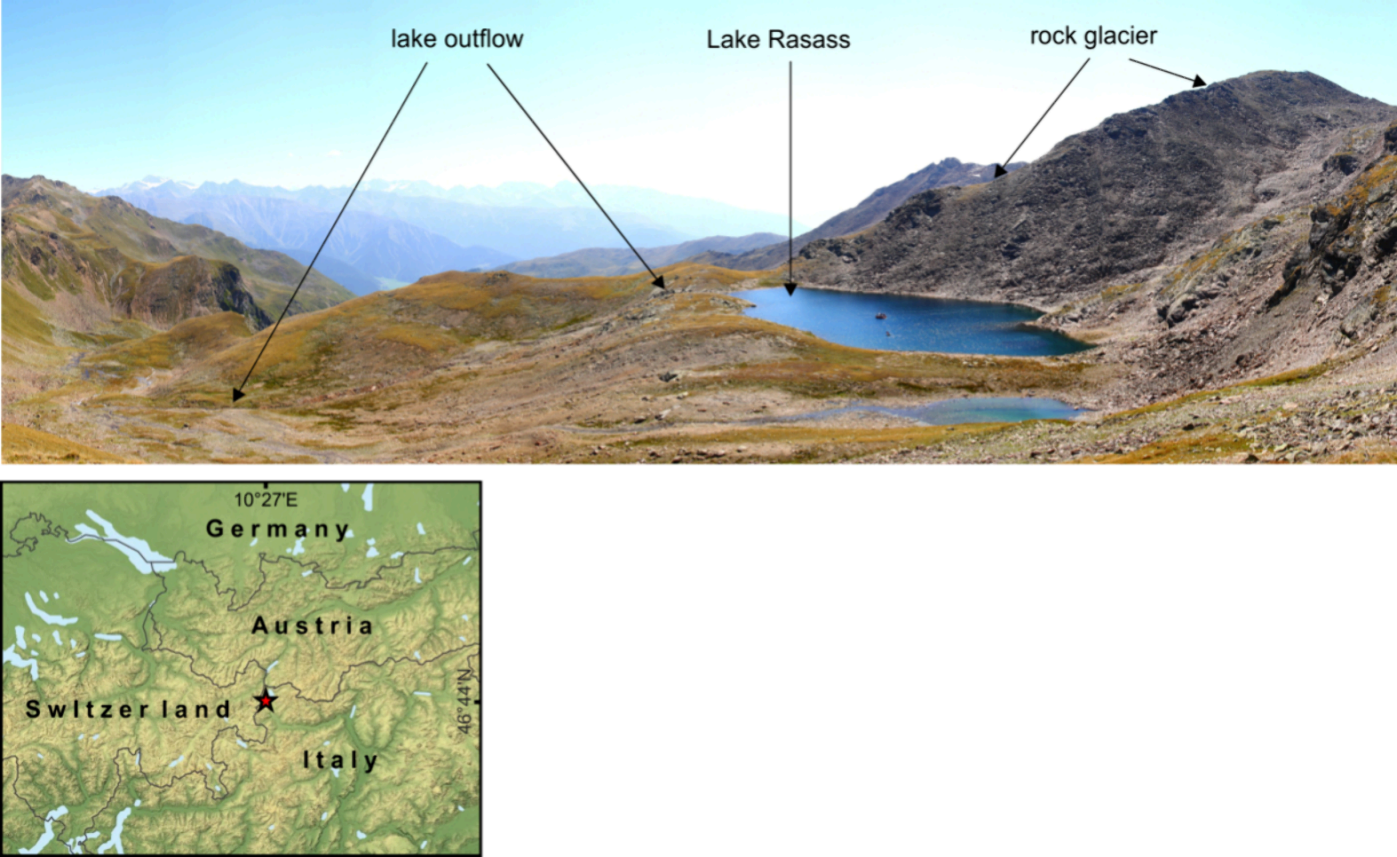
Study catchment and its location on the map.

Rock samples for the series of leaching experiments were collected in the catchment area of Lake Rasass, an alpine headwater lake (2682 m a.s.l.) situated beneath an intact rock glacier (2700–2800 m a.s.l.) in the Italian Alps, near the Austrian and Swiss borders (Figure 1). The location of the lake with a maximum depth of 9.3 m at the toe of the intact rock glacier points to its close connection with the internal drainage network of the rock glacier. The groundwater from the rock glacier lacking discharge in the form of streams or springs is discharged directly into the lake. The catchment outflow is presented by a stream flowing from the lake. Previous studies have shown that the catchment hosts NARD generated by the rock glacier.^24,25^ In the lake receiving solutes from the rock glacier, the acidic water (pH 5.1–5.5) is characterized by extremely high concentrations of dissolved sulfate, aluminum, and trace metals such as manganese and nickel.^24,25,33^ Abnormal levels of these solutes in the lake are beyond surface water quality standards and pose potential health and environmental risks. Of particular concern are the high concentrations of nickel (240–300 μg L^–1^) that has a very high toxicity and carcinogenicity to humans and other living organisms.^34^ The high concentrations of manganese (560–1200 μg L^–1^) also represent a serious health hazard to humans, resulting in severe pathologies of the central nervous system.^35^ A previous study involving sedimentological and paleo-ecotoxicological research techniques has provided evidence of the long-lasting nature of the NARD that took place in the early Holocene and during at least the last 2.5 millennia.^24^

Geologically, the study area belongs to the polymetamorphic Oetztal-Stubai crystalline complex that is mainly composed of silica-rich rocks, namely, gneiss and schist with intercalations of quartzite, amphibolite, eclogite, and marble.^36^ Sulfide minerals are ubiquitous in these metamorphic rocks, but they are commonly present in small amounts as accessory minerals.^14,37,38^ The study catchment in particular is underlain by crystalline rocks, mainly gneiss (paragneiss and orthogneiss) and schist, and as revealed through X-ray diffraction and Rietveld analysis, the rock-forming minerals are quartz (50%), feldspar (27%), muscovite (15%), chlorite (6%), and dolomite (2%).^33,39^ The chemical composition of the bedrock is characterized by concentrations of nickel and manganese of ∼25–30 and ∼600–775 μg g^–1^, respectively, that do not exceed their abundances in the Earth’s continental crust.^33,39^

For subsequent leaching experiments, a representative sample of paragneiss (∼20 kg) was obtained by combining rock debris collected randomly from nonglaciated parts of the catchment. Only rock fragments with the *a*-axis (longest axis of 3D objects) of 35–40 mm and without visible evidence of weathering were used in the study. This size fraction was taken as an intermediate one for a mixture of fine and coarse rock fragments, with a size range of <1 mm to >1 m,^40^ incorporated into the body of rock glaciers. The sample was transported to the laboratory, where the rock fragments were rinsed with deionized water and air-dried. Thereafter, the bulk sample was split into replicate samples of 2.2 kg (105–120 rock fragments). In addition, three randomly selected paragneiss specimens from the bulk sample were used for the subsequent geochemical analysis.

### 2.2 Laboratory Experimental Design

**Figure 2:**
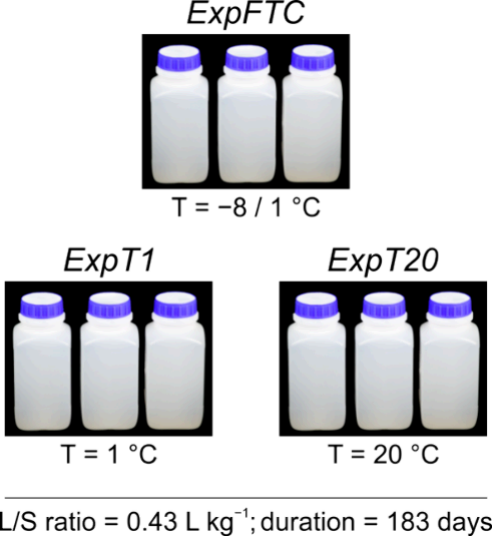
Sketch showing the overall design of the experiments.

For each of the three separate experiments described below, three replicate samples of rock fragments were placed into separate, acid washed 1,800 mL high-density polyethylene (HDPE) wide-mouth bottles, then covered with 950 mL of Milli-Q deionized water (L/S ratio = 0.43 L kg^–1^), and stored upright at temperatures specified below to interact for 183 days (∼26 weeks) (Figure 2). The mouth of the bottles was sealed with the filter paper to provide access to oxygen. The pH was measured every 48 h during the first 2 weeks and every 2 weeks for the rest of the time. The bottles were shaken by hand for 15 s every 2 weeks after the pH measurements during the course of the experiments. No sampling of the leachates for chemical analysis was carried out to keep the liquid/solid ratio constant during the course of the experiments.

At the end of the experiments, the leachates were filtered through Sartorius cellulose acetate membrane filters (0.45 μm pore diameter) into 500 mL HDPE bottles for the analysis of major cations and anions. Additional aliquots of the filtrates were collected into 100 mL HDPE bottles and acidified with 1 mL of ARISTAR grade HNO_3_ for subsequent analysis of dissolved metals (Al, Ba, Cd, Co, Cr, Cu, Fe, Mn, Ni, Pb, Si, Sr, and Zn) and silicon. In addition, aliquots of the resultant leachates after diurnal freeze–thaw cycles were acidified with 5 mL of 1 M HNO_3_ before the filtration to convert all nonreactive silica species to reactive species and to desorb colloid-bound trace elements. All bottles used in the study were previously soaked in 5% HNO_3_ for at least 24 h and then thoroughly rinsed with deionized water.

Rock particles smaller than 35 mm (fine-grained material) appearing during the course of the experiments were attributed to chemical and physical weathering, two overlapping types of processes typically involved in the rock disintegration.^41^ Upon completion of the experiments, the fine-grained material accumulated at the bottom of each bottle was dried until constant dry weight at 50 °C in a laboratory furnace and weighed with an accuracy of 0.01 g using an electronic balance before grain size analysis.

- Experiment 1 (ExpT1): rock disintegration and solute release at a temperature just above the freezing point of water. The samples were incubated at 1 °C. This temperature was selected as an average value over a temperature range from 0 to 2 °C since generally groundwater discharging from intact rock glaciers (i.e., with permafrost ground ice) is maintained within this temperature range throughout the whole year.^40,42^
- Experiment 2 (ExpFTC): rock disintegration and solute release under long-term freeze–thaw conditions. A second set of experiments was subjected to repeated diurnal FTCs between −8 and 1 °C using a freezer, refrigerator, and fan linked to automatic timers. The samples were cooled to −8 °C for 6 h and maintained at this temperature for a further 6 h. Then, the samples were warmed until 1 °C for 6 h, followed by this temperature of 6 h. The applied range in temperature was selected because it encompasses a window between −3 and −8 °C termed “frost cracking window”, which is the temperature range in which frost cracking of most rocks caused by ice segregation is significantly enhanced.^43,44^ Diurnal FTCs can be widespread within the uppermost ∼50 cm of bedrock and rock debris during all seasons except midsummer.^45−47^
- Experiment 3 (ExpT20): rock disintegration and solute release at a reference temperature of 20 °C. The samples were incubated at 20 °C. Since it is known that the dissolution rate of minerals is strongly temperature-dependent and increases with temperature,^13,18^ this temperature, not typical for rock glaciers, was only used as a reference temperature for comparison and subsequent analysis. The data from ExpT20 were compared to the results from ExpT1 and ExpFTC.

### 2.3 Analytical Methodology

The chemical composition of the paragneiss samples (fine-grinded powder in Chemplex SpectroMicro Sample Cups 3110) was assayed by energy-dispersive X-ray fluorescence (ED-XRF) analysis using a Spectro Xepos Plus spectrometer and calibration models based on the fundamental parameter methods.^48^ Calibrations of accuracy and reproducibility were conducted using the CCRMP reference soils.^49^ The relative standard deviation obtained by the method of standard additions is ±10% for oxides of major elements and ±2–5% for all others.

The distribution of the fine-grained rock material accumulated at the bottom of bottles into size fractions of <0.125, 0.125–0.250, 0.25–0.50, 0.5–2.0, 2–10, and >10 mm was derived by sieving the material through Endecotts laboratory test sieves using a sieve shaker (Endecotts Minor 200) for 15 min.

The pH of the leachates was measured using a pH meter (LAQUA PH210, Horiba) with a built-in temperature sensor for temperature compensation of the pH values to a reference temperature of 25 °C, calibrated at pH 7.00 and 4.00 using commercial pH buffers. The chemical composition of the leachate samples was analyzed following standard techniques.^50^ Bicarbonate ions were quantified by acidimetric titration and all other major ions by ion chromatography (Dionex DX-120 Ion Chromatograph) at the University of Innsbruck, Austria. The concentrations of dissolved metals and silicon were determined by inductively coupled plasma-optical emission spectrometry (Thermo Scientific iCAP 6500 Duo Spectrometer) at the laboratory of the Alice Holt Research Station, UK. Standard analytical quality control protocols were observed for the analysis, including blanks and initial calibration verification by certified standards for each element, continuing calibration verification every 10 samples, and a dilution check. Total dissolved solids (TDS) were calculated using AquaChem software (Waterloo Hydrogeologic Inc.).

### 2.4 Statistical Analysis

One-way analysis of variance (ANOVA), followed by Tukey’s post hoc test, was used to determine significant differences in properties of the fine-grained rock materials and the leaching solutions formed through the experiments to find the effects of temperature and the presence or absence of FTCs on rock disintegration as well as on acid generation and the release of trace elements. All data were transformed using the base 10 logarithm to reduce the biasing effect of extremely high or low values.

## 3 Results and Discussion

### 3.1 Geochemistry of Paragneiss

Concentrations of major-element oxides, SiO_2_ of 62.6 wt % and Al_2_O_3_ of 15.6 wt %, are typical of gneisses formed by metamorphic processes from sedimentary rocks.^51,52^ The concentrations of most trace elements are lower than or comparable to their abundance in the upper continental crust (Table 1). The average chlorine content, for example, is slightly decreased (177 μg g^–1^) relative to its abundance in the upper continental crust (370 μg g^–1^). The average chlorine content for gneisses is ∼200 μg g^–1^,^54^ where Cl can reside in halite-bearing (NaCl) fluid inclusions,^52,55^ and, besides, accessory minerals such as hornblende, biotite, and apatite can incorporate Cl into their crystal structure.^52,56^ In addition, in some silicates, for example, sodalite and scapolite, Cl is a major constituent.^57^ The sulfur, nickel, and manganese content is comparable to their abundance in the upper continental crust (Table 1). An extensive data set from the Eastern Alps, including the Oetztal-Stubai crystalline complex,^38^ provides evidence that nickel is preferentially incorporated into the crystal structure of micro- to nanosized inclusions of sulfide minerals, namely, pyrite and pyrrhotite. Manganese within this area is usually hosted in sphalerite,^38^ another sulfide mineral that in turn is commonly associated with pyrite and/or pyrrhotite.^58^

**Table 1:** Chemical Composition of Paragneiss in the Study Area and the Average Abundances of the Elements in the Upper Continental Crust

component	this work (*n* = 3)	LLD^a^	upper continental crust^b^
Oxides of major elements (wt %)			
SiO_2_	62.58	0.0005	66.62
Al_2_O_3_	15.59	0.0005	15.40
FeO_T_^c^	5.87	0.0002	5.04
MnO	0.09	0.0001	0.10
MgO	2.14	0.0010	2.48
CaO	1.60	0.0007	3.59
Na_2_O	2.58	0.0050	3.27
K_2_O	2.64	0.0004	2.80
TiO_2_	0.77	0.0006	0.64
P_2_O_5_	0.11	0.0001	0.15
Trace elements (mean ± SD)^d^ (μg g^–1^)			
As	5 ± 2	1	5
Ba	1065 ± 688	20	624
Cl	177 ± 15	10	370
Cr	94 ± 30	2	92
Cu	31 ± 23	1	28
Ga	22 ± 7	1	18
Hf	8 ± 1	3	5
Nb	16 ± 3	2	12
Ni	48 ± 15	1	47
Pb	21 ± 4	2	17
Rb	95 ± 36	1	84
S	617 ± 40	20	621
Sr	252 ± 35	1	320
Th	15 ± 5	2	11
V	106 ± 33	3	97
Y	31 ± 3	1	21
Zn	86 ± 11	1	67
Zr	270 ± 41	2	193

a LLD = lower limit of detection.

b From Rudnick and Gao.^53^

c Total Fe as FeO.

d Concentrations of Ag, Bi, Br, Cd, Ge, I, Mo, Sb, Se, Sn, Ta, Tl, U, and W are below LLDs for each of them.

### 3.2 Rock Disintegration

**Figure 3:**
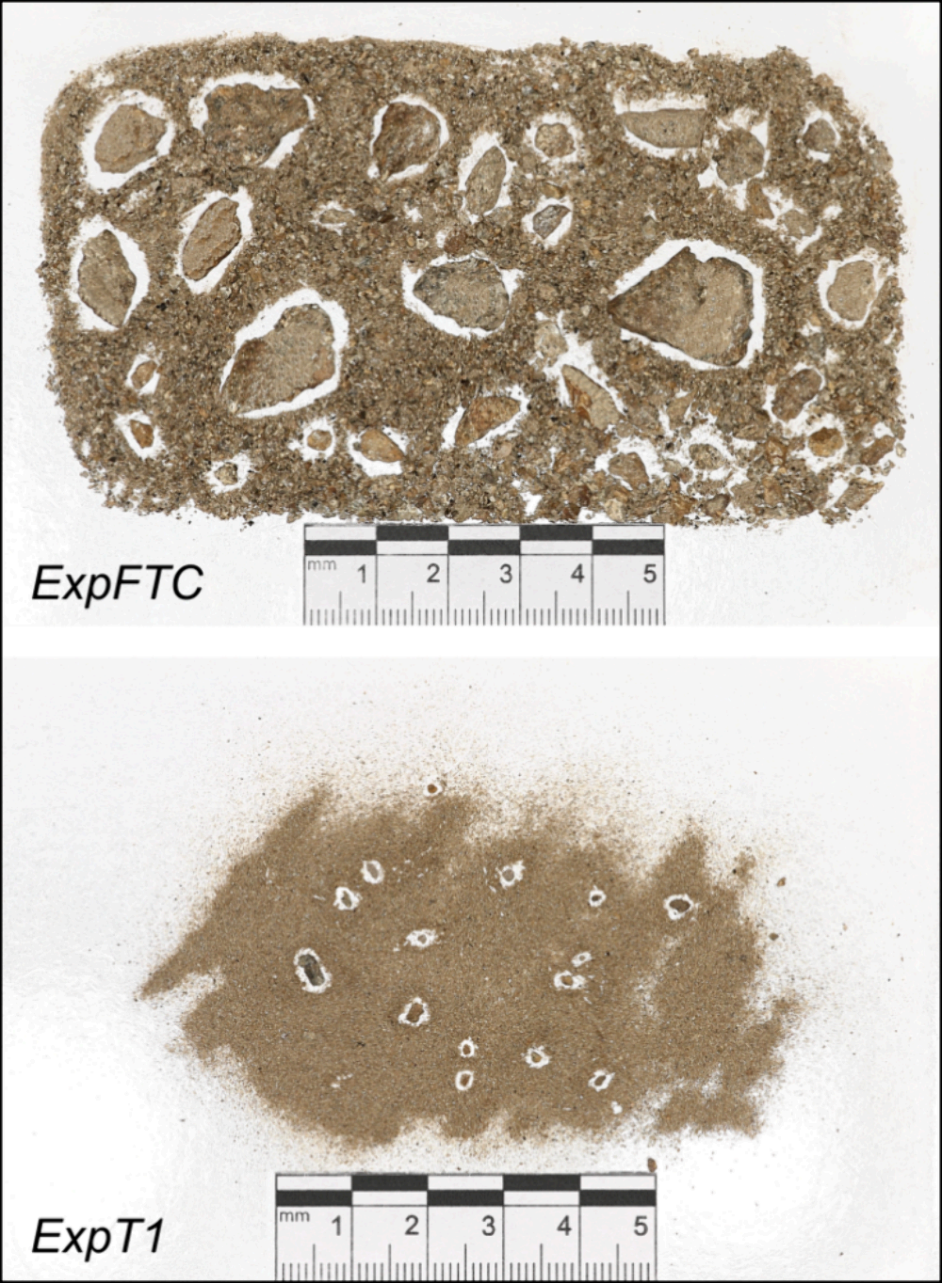
Weathered fine debris from paragneiss by the end of leaching experiments: ExpFTC—under the action of diurnal freeze–thaw cycles (−8/1 °C) and ExpT1—at a temperature of 1 °C.

By the end of the experiments, the samples subjected to FTCs (ExpFTC) have produced the largest amounts of fine-grained debris that ranges in size from <0.125 to 20 mm, whereas weathering processes at 1 and 20 °C (ExpT1 and ExpT20) yielded much lesser amounts of fine-grained debris that is mainly in the less than 0.25 mm size range (Table 2 and Figure 3). Weathering processes in the absence of FTCs were 6–7 times less effective in transferring material into the fine-grained fraction, compared with ExpFTC (*p* < 0.05, Tukey’s post hoc test). These differences may have been due to differences in the set of weathering processes operated during the course of each of the experiments.

**Table 2:** Amount and Particle Size Distribution of Fine Debris (<0.125–20 mm) Produced by the Leaching Experiments

experiment	total mass (g)^a^	particle size fraction (%)					
		<0.125 mm	0.125–0.250 mm	0.25–0.50 mm	0.5–2.0 mm	2–10 mm	>10 mm
ExpFTC	35.3 ± 1.9^a^	6.1	11.5	18.7	15.0	14.4	34.3
ExpT1	5.2 ± 0.4^b^	42.0	39.5	15.2	0.9	2.4	0.0
ExpT20	6.0 ± 0.9^b^	43.8	42.1	13.2	1.0	1.5	0.0

a Mean ± SD, *n* = 3; within the column, means with different superscripts are statistically different from each other (*p* < 0.05, Tukey’s post hoc test).

The processes of chemical weathering, such as dissolution of minerals (see the next section for details), occurred in all the experiments and resulted in the weakening and subsequent granular disintegration of the rock. The processes of physical weathering resulting in production of the fine debris <0.5 mm in all the experiments also included abrasion in addition to granular disintegration. This occurred every time the rock fragments scraped against each other due to shaking the samples.

Enhanced grain detachment and exfoliation, the processes of physical weathering leading to an increased production of the fine debris (μm-to-cm scale) in ExpFTC, may be explained by the effects of repeated FTCs. Freeze–thaw action, also referred to as frost weathering, is the dominant physical process operating wherever rocks experience temperature fluctuations across the freezing point and moisture is present.^59^ Frost weathering processes comprise (1) volumetric expansion generated by the 9% volumetric increase when water changes phase to ice in rock pores and cracks, which takes place under conditions of rapid freezing and significant temperature reduction below the freezing point; and (2) ice segregation, occurring when temperature gradient-induced suction drives water migration through a porous medium toward freezing sites where lenses of segregated ice grow.^59,60^ In the case of ExpFTC, slow cooling to −8 °C for 6 h and a constant temperature of −8 °C for the next 6 h were provided sufficient time for water migration to the freezing front and favored frost weathering by ice segregation. The theory of ice segregation suggests that cracks in intact, low-porosity rocks (like gneiss) propagate at temperatures below −4 °C,^61,62^ but the presence of initial microcracks in gneiss can favor ice segregation within a temperature range of −0.5 to −2.7 °C.^63^

The obtained results suggest that the changes in phase from liquid water to solid ice and vice versa played an important role in the disintegration of the rock due to segregation freezing and in the production of fine debris with fresh, reactive mineral surfaces susceptible to chemical weathering. The particle size reduction and the origins and development of microcracks undoubtedly increased the specific surface area of the rock fragments^64,65^ and further facilitated chemical weathering.^41,66^ Disintegration of rocks due to ice segregation is likely one of the main processes, leading to an abundance of fine debris. This debris as a result of washing away and kinetic sieving can migrate downward^67^ and contribute to the formation of a ∼ 10 m-thick unfrozen, fine-grained sediment layer that is typically detected between the bedrock and the permafrost body of intact rock glaciers.^11,68^ A low hydraulic conductivity of such fine-grained sediments facilitates a relatively long residence time of groundwater and enhanced chemical weathering of freshly exposed rock surfaces.

### 3.3 Leachate Geochemistry

#### 3.3.1 Leaching of Major Ions

The average TDS values of the resultant leachates were 42 mg L^−1^ in ExpFTC, 56 mg L^−1^ in ExpT1, and 75 mg L^−1^ in ExpT20. Due to the very low solubility of silicate minerals, similar low amounts of total dissolved solids are typically characteristic for groundwater from shallow crystalline aquifers.^12,14^ In addition, the low temperatures in ExpFTC and ExpT1 undoubtedly exerted kinetic limitations on chemical weathering. Given that the interaction between liquid water and rock under the freeze–thaw conditions was half as long as in ExpT1, one would expect the efficiency of water–rock interactions in ExpFTC to be half as low as in ExpT1. However, no statistically significant differences in the amounts of total dissolved solids were found between ExpFTC and ExpT1 (*p* > 0.05, Tukey’s post hoc test). The expected effect of the shortened duration of water–rock interactions in ExpFTC has likely been offset by the increased fracture-induced exposure of fresh rock surfaces with unweathered minerals.

**Figure 4:**
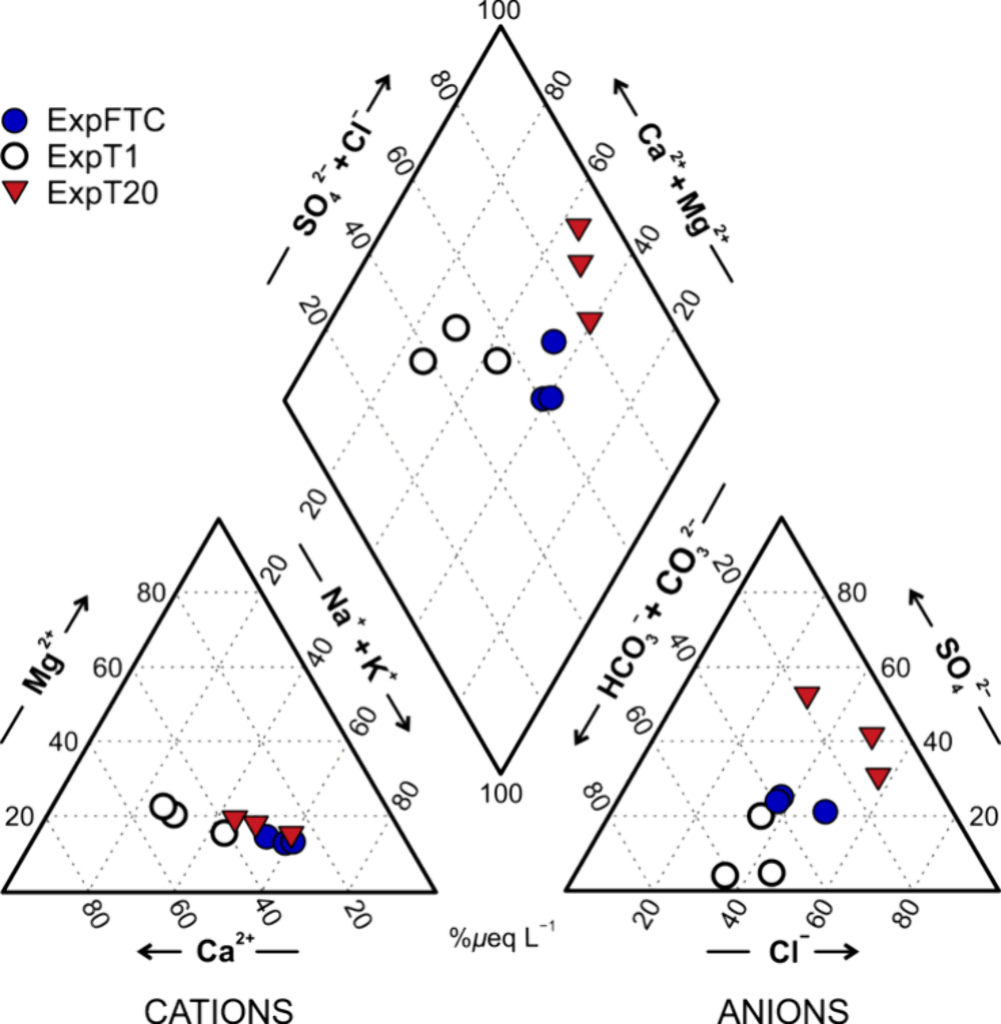
Trilinear diagram showing the differences in geochemistry of the resultant leachates. The leaching experiments: ExpFTC—under the action of diurnal freeze–thaw cycles, ExpT1—at a temperature of 1 °C, and ExpT20—at a temperature of 20 °C.

The proportions of the major solutes acquired by water–rock interaction differed between the experiments (Figure 4 and Table S1). The cation compositions of the resultant leachates, in terms of percentages in equivalent units, were dominated by Na^+^ and Ca^2+^ in all three experiments, but in different proportions: Na^+^ > Ca^2+^ (44 and 22%, respectively) in ExpFTC, Ca^2+^ > Na^+^ (39 and 22%, respectively) in ExpT1, and Na^+^ > Ca^2+^ (35 and 26%, respectively) in ExpT20. The differences in the anion compositions of the leachates were more pronounced. The leachates in ExpFTC and ExpT1 were dominated by Cl^–^ and HCO_3_^–^ in different proportions: Cl^–^ > HCO_3_^–^ (41 and 32%, respectively) in ExpFTC and HCO_3_^–^ > Cl^–^ (50 and 39%, respectively) in ExpT1. The ExpT20 leachates were dominated by Cl^–^ (37%) and SO_4_^2–^ (35%).

The chemical composition of the leachates, especially in ExpFTC and ExpT20 where sodium was the most abundant cation and chloride the most abundant anion, is uncommon for groundwater from crystalline aquifers^69^ but is found in local areas where leaching of halite-bearing (NaCl) fluid inclusions is a source of salinity.^52,70−72^ Leaching experiments by Bucher and Stober,^52^ Waber and Smellie,^73^ and Seelig and Bucher^74^ have shown that a considerable amount of chloride could be readily mobilized from rocks where it is present as halite in fluid inclusions. The available data suggest that leaching of metamorphic fluids from the pore space of the crystalline rock matrix was the main source of Cl in our experiments, although other sources cannot be excluded. The lack of measurement data on bromide in this study did not allow us to use the Cl/Br ratio as a tracer to identify with confidence the origin of Cl.

**Figure 5:**
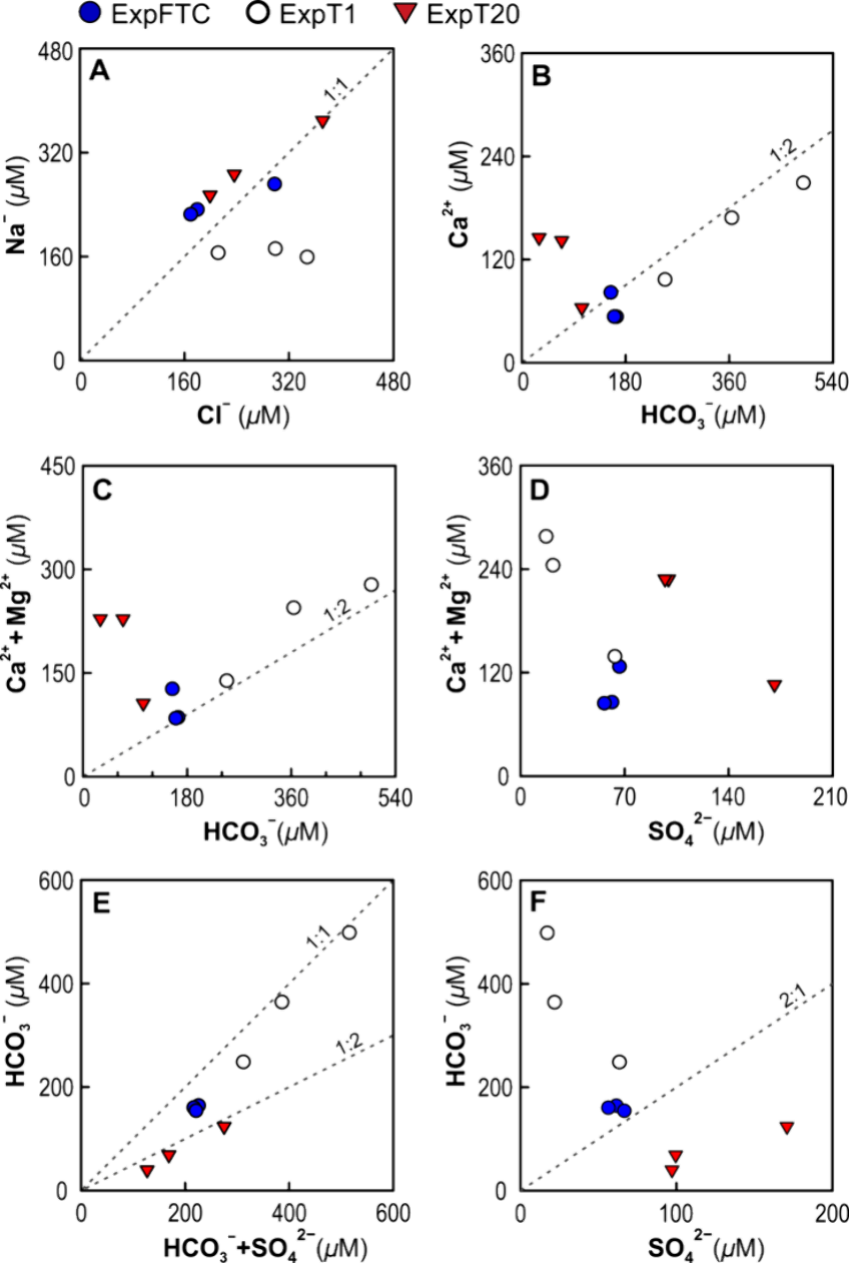
Molar Na^+^ concentrations compared to Cl^–^ in the resultant leachates (A), as well as Ca^2+^ vs HCO_3_^–^ (B), Ca^2+^ + Mg^2+^ vs HCO_3_^–^ (C), Ca^2+^ + Mg^2+^ vs SO_4_^2–^ (D), HCO_3_^–^ vs HCO_3_^–^ + SO_4_^2–^ (E), and HCO_3_^–^ vs SO_4_^2–^ (F). See the text for an explanation of the 1:2, 1:1, and 2:1 ratios. The leaching experiments: ExpFTC—under the action of diurnal freeze–thaw cycles, ExpT1—at a temperature of 1 °C, and ExpT20—at a temperature of 20 °C.

Molar Na^+^/Cl^–^ ratios were predominantly close to 1.0, or in some cases <1, in all leachates (Figure 5A). Therefore, sodium and chlorine may have been derived primarily from leaching of NaCl-rich, like halite-bearing, fluid inclusions, whereas dissolution of Na-silicates, commonly resulting in Na^+^/Cl^–^ ratios of >1.0,^72^ provided a negligible contribution of chlorine. In all leachates, HCO_3_^–^ and Ca^2+^ were likely derived primarily from chemical weathering of Ca-bearing silicates and disseminated calcite.^75^ This is confirmed by the molar ratios of Ca^2+^ to HCO_3_^–^ and (Ca^2+^ + Mg^2+^) to HCO_3_^–^ that are distributed around the 1:2 line (Figure 5B,C). At a temperature of 20 °C in ExpT20, these ratios were predominantly far larger than 0.5, indicating a greater contribution of the silicate dissolution resulting in the relative excess of Ca^2+^ and Mg^2+^.^76^ Molar Ca^2+^/Mg^2+^ ratios >1 (1.6–2.2) in all leachates also indicate the silicate and calcite dissolution, whereas the ratios of around 1.0 can signify the dissolution of dolomite [CaMg(CO_3_)_2_].^77^ The lack of correlation between (Ca^2+^ + Mg^2+^) and SO_4_^2–^ (Figure 5D) signifies that SO_4_^2–^ was not contributed to the leachates by the dissolution of sulfate minerals but was originated from the oxidative dissolution of sulfide minerals.^12,72^ Molar HCO_3_^–^/(HCO_3_^–^ + SO_4_^2–^) ratios were about 0.9 in the ExpT1 leachates and between 0.4 and 0.7 in the ExpFTC and ExpT20 leachates (Figure 5E). Brown et al.^78^ showed that the ratio of HCO_3_^–^ to (HCO_3_^–^ + SO_4_^2–^) of 1.0 signifies the carbonate dissolution, whereas the ratios less than 1.0 suggest the coupled sulfide oxidation and carbonate dissolution. Molar HCO_3_^–^/SO_4_^2–^ ratios in the ExpT1 leachates were far larger than 2, suggesting that carbonate rather than sulfide dominates (Figure 5F).

The obtained results suggest that dissolution of halite-rich fluid inclusions and the coupled reactions of sulfide oxidation and carbonate dissolution are the most important processes that control solute acquisition by water–rock interactions within the rock glacier, both at a temperature of 1 °C and under freeze–thaw conditions. The amount and proportion of the major solutes acquired by water–rock interactions are most likely controlled by the availability of minerals with fast reaction kinetics, the reactive mineral surface area, and temperature. Carbonate and sulfide minerals dissolve up to ∼3 orders of magnitude faster and chlorides, including halite, up to ∼15 orders of magnitude faster than the kinetically sluggish silicates.^79−81^ In our experiments, carbonate and sulfide minerals and halite-rich fluid inclusions, which are present as trace constituents of the silicate rock, largely determined the major element composition of the leachates. Thus, based on the results of the investigation, we could deduce with high certainty that these minerals with fast reaction kinetics contribute the major proportion of solute in groundwater within the rock glacier as well.

#### 3.3.2 Evolution of Leachate pH

**Figure 6:**
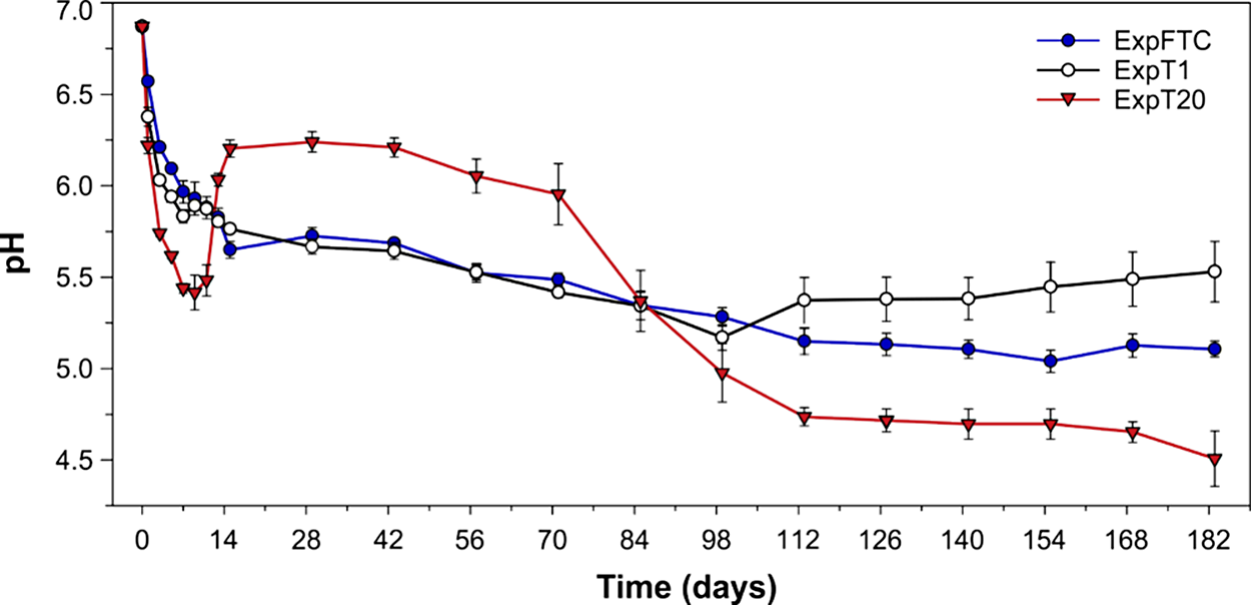
Changes in pH (mean ± SD, *n* = 3) over time during the leaching experiments: ExpFTC—under the action of diurnal freeze–thaw cycles, ExpT1—at a temperature of 1 °C, and ExpT20—at a temperature of 20 °C.

As shown in Figure 6 and Table S2, the pH of the leachates decreased from the original leaching solution value of ∼6.9 to 5.8–6.0 in ExpFTC and ExpT1 and to 5.4 in ExpT20 during the initial 7 days. This implies that easily dissolved chemical species, including sulfides, were released promptly after the addition of deionized water. Afterward, the observed patterns of pH changes differed between the experiments. In ExpFTC, the leachate pH had a gradually decreasing trend up to 5.0–5.1 during the next ∼105 days and started to level off after that. In ExpT1, the leachate pH gradually decreased and reached the lowest value of around 5.2 after ∼98 days. Then, it increased slightly, by ∼0.3 pH unit, by the end of the experiment. In ExpT20, the initial downward trend in the pH values was replaced by an increase to 6.0–6.2 during the next 7 days. These values were maintained for ∼55 days and then began to decrease to the lowest values at the end of the experiment. The increased pH values in the course of the first half of ExpT20 (Figure 6) were likely attributed to an enhanced chemical weathering of trace carbonate minerals, the dissolution rates of which tend to increase with increasing temperature within the range of 5–25 °C.^65^ After ∼70 days of the experiment, the most accessible part of the carbonate minerals has been dissolved, and the pH values began to decrease.

Thus, the pH values were within the range of acidic conditions throughout all the experiments, revealing a very low acid neutralization capacity of the rock. As mentioned above, the dissolution of sulfide minerals was the main source of dissolved sulfate and likely the most important process that controls acidity. At the end of the experiments, the average pH values followed the order of ExpT1 (5.5) > ExpFTC (5.1) > ExpT20 (4.5) (*p* < 0.05, Tukey’s post hoc test). The order likely reflects kinetic-limited sulfide dissolution at low temperatures in ExpT1 and ExpFTC and also the increased fracture-induced exposure of fresh rock surfaces, leading to enhanced sulfide dissolution in ExpFTC compared with that in ExpT1. Accordingly, the average concentrations of SO_4_^2–^ in the resultant leachates were in the opposite order: ExpT1 (68 μeq L^–1^) < ExpFTC (123 μeq L^–1^) < ExpT20 (245 μeq L^–1^). These results are consistent with the study by Elberling,^82^ who found that oxidation of sulfide minerals is not reduced to negligible levels at 1 °C. Furthermore, leaching experiments by Ethier et al.^83^ have shown that the sulfur leaching from sulfide minerals can be 1.5–2 times higher when FTCs are applied, even in comparison to the rates at 22 °C.

#### 3.3.3 Leaching of Trace Elements

The oxidation of sulfide minerals is an important process for the mobilization of aquifer lithology-specific trace elements in groundwater. Once mobilized, they are generally subject to solubility control and/or surface interactions (adsorption or coprecipitation) with other solid fractions.^84^ The solubility and mobility of many trace elements, especially metals, are strongly affected by pH conditions, resulting in their highly elevated concentrations in acidic waters.^18^ In addition, the leaching rates of many heavy metals increase as temperature rises.^85^

The temporal change patterns of pH obtained in all of the experiments indicate acidic conditions facilitating the solubility and mobility of trace elements for the entire duration of the experiments (Figure 6). The concentrations of Al, Ba, Co, Mn, Ni, Sr, and Zn in the resultant leachates of ExpT20 were significantly greater (*p* < 0.05, Tukey’s post hoc test) than in the less acidic ones of ExpT1 (Table 3). Particularly noteworthy are the concentrations of potentially toxic manganese and nickel in the ExpT20 leachates, which exceeded the appropriate European Union standards for water intended for human consumption^85^ more than 43 and 5 times, respectively. The concentrations of Mn and Ni in the ExpT1 leachates were also high and exceeded the appropriate EU standards more than 26 and 2 times, respectively, which may confirm a geogenic origin of these elements in the groundwater of the rock glacier and in the discharge lake and do not support the hypothesis of their technogenic and/or volcanic origin from atmospheric deposition in the ice matrix of rock glaciers.^87,88^ Such elevated concentrations of trace elements, forming in groundwater through purely natural processes, are not an uncommon phenomenon and represent natural background concentrations for shallow groundwater in a given region.^12,14^

**Table 3:** Concentrations (μg L^–1^; Mean ± SD, *n* = 3) of Dissolved Trace Elements and Silicon in the Leachates by the End of Experiments: ExpFTC_D_ and FTC_T_—under the Action of Diurnal Freeze–Thaw Cycles, ExpT1—at a Temperature of 1 °C, and ExpT20—at a Temperature of 20 °C; ExpFTC_D_—after Filtration, and ExpFTC_T_—after Treatment with HNO_3_ and Subsequent Filtration^a^

	experiment				LLD	EU limit value
	ExpFTC_D_	ExpFTC_T_	ExpT1	ExpT20		
Al	16 ± 3^*a*^	30 ± 5^*b*^	3 ± 3^*a*^	39 ± 9^*b*^	0.07	200
Ba	10 ± 3^*a*^	29 ± 5^*b,c*^	18 ± 4^*a,c*^	41 ± 7^*c*^	0.06	n/a
Cd	<LLD	<LLD	<LLD	0.4 ± 0.3	0.10	5
Co	0.3 ± 0.2^*a*^	28 ± 3^*b*^	19 ± 7^*c*^	35 ± 5^*b*^	0.10	n/a
Cr	<LLD	<LLD	<LLD	<LLD	0.10	25
Cu	0.9 ± 0.2^*a*^	2.2 ± 0.7^*a,b*^	1.7 ± 0.4^*a*^	3.7 ± 1.0^*b*^	0.50	2000
Fe	5 ± 3^*a*^	4 ± 3^*a*^	3 ± 3^*a*^	4 ± 4^*a*^	0.60	200
Mn	27 ± 9^*a*^	**1862** ± **235**^*b*^	**1331** ± **158**^*c*^	**2199** ± **246**^*b*^	0.08	50
Ni	10 ± 1^*a*^	**79** ± **10**^*b*^	**44** ± **4**^*c*^	**116** ± **14**^*d*^	0.10	20
Pb	<LLD	0.6 ± 0.3^*a*^	0.5 ± 0.3^*a*^	1.3 ± 0.3^*a*^	0.20	5
Si	194 ± 67^*a*^	3970 ± 503^*b*^	3080 ± 328^*b*^	7322 ± 379^*c*^	2.30	n/a
Sr	27 ± 4^*a*^	54 ± 3^*b*^	57 ± 3^*b*^	72 ± 4^*c*^	0.01	n/a
Zn	26 ± 4^*a*^	47 ± 4^*a*^	26 ± 2^*a*^	136 ± 22^*b*^	0.14	n/a

a *^a–c^*Within a row, means with different superscripts are statistically different from each other (*p* < 0.05, Tukey’s post hoc test); values exceeding the appropriate European Union limit values for water intended for human consumption^86^ are shown in bold; LLD = lower limit of detection; n/a = not available.

At the first glance, the extremely low concentrations of the trace elements in the ExpFTC leachates after filtration (hereinafter as ExpFTC_D_ leachates), despite the pH values of these leachates being 0.4 units lower than in ExpT1, might appear rather unexpected. However, it has been shown that freezing and FTCs may induce an enhanced polymerization of dissolved silica originating from the dissolution of rocks to amorphous colloidal silica (SiO_2_).^89,90^ This amorphous solid represents a highly effective adsorbent for metal ions in aqueous solution^89,91,92^ and a vehicle for their enhanced transport in groundwater.^93,94^ In our case, the dispersed amorphous silica with colloid-bound trace elements formed under the cyclic freezing conditions was removed from the ExpFTC_D_ leachates by filtration through a 0.45 μm filter. This is consistent with the extremely low concentrations of dissolved silicon in the ExpFTC_D_ leachates (Table 3) and with a high abundance of a clear jelly-like substance on the surface of the filters after filtration.

Thereafter, other aliquots of the ExpFTC leachates have been treated with HNO_3_ before the filtration (hereinafter as ExpFTC_T_ leachates) to convert all nonreactive (colloidal) silica species to reactive (soluble) species and to desorb colloid-bound trace elements. The total silicon concentrations (from nonreactive and reactive silica) in the ExpFTC_T_ leachates suggest that the dissolved silicon concentrations (from reactive silica) accounted for only 5% of them just after melting of the ExpFTC leachates (Table 3). The total concentrations of many trace elements (Al, Ba, Co, Mn, Ni, and Sr) in the ExpFTC_T_ leachates were also significantly greater (*p* < 0.05, Tukey’s post hoc test) than in the ExpFTC_D_ leachates. It confirms the fact that many metal ions were removed effectively from the ExpFTC_D_ leachates by adsorption onto amorphous silica. Meanwhile, the concentrations of Mn and Ni in the ExpFTC_T_ leachates were 1.4 and 1.8 times, respectively, higher than those in the resultant leachates of ExpT1. Apparently, leachates enriched with amorphous silica and colloid-bound metals from the wet upper and ice-rich lower layers of rock glaciers, where diurnal, seasonal, and/or interannual FTCs occur, migrate downward as a result of washing away and redissolution of amorphous silica within unfrozen layers and may represent an additional source of trace elements.

#### 3.3.4 pH and Trace Elements: Leachates vs Lake Water

The pH values of the resultant leachates in ExpFTC (5.1) and ExpT1 (5.5) were comparable to the seasonal variation in water pH (5.1–5.5) of Lake Rasass, a discharge lake receiving groundwater from the rock glacier.^24,25,33^ Concentrations of Mn (1330–1860 μg L^–1^) and Ni (45–80 μg L^–1^) obtained in the ExpFTC and ExpT1 leachates were also roughly comparable to their seasonal variation in the lake water (560–1200 and 240–300 μg L^–1^, respectively).^24,25,33^ Unfortunately, further more detailed comparisons of the leachate and lake water geochemistry may be unjustified since the chemical composition of water in the lake is controlled also by many other processes such as atmospheric precipitation, water surface evaporation, soil formation and erosion in the catchment, etc.

### 3.4 Advantages and Limitations of the Research

The experimental method used has benefits and limitations that must be considered. The advantages of laboratory experiments are their reproducibility and, especially, the option to investigate each parameter separately. Under natural conditions, some other factors may exacerbate NARD, and therefore, the findings of this study should be considered in light of some limitations of the experimental conditions. First, besides diurnal FTCs when the freezing front can reach ∼50 cm depth in rock,^45−47^ rock fragments incorporated into the body of rock glaciers can also be impacted by seasonal FTCs. During the cold season, longer periods of subzero temperatures can be reached, which favor intensive rock disintegration caused by ice segregation at depths up to ∼5 m.^45,62^ Second, volumetric expansion upon rapid freezing and significant temperature reduction, particularly at south-facing sites,^45,47^ can also lead, along with ice segregation, to the production of fine debris with freshly exposed rock surfaces. Furthermore, freezing in nature occurs often in combination with wetting and drying, and additional stresses caused by wetting-drying cycles can superpose the effects of ice segregation and volumetric expansion.^41^ Further limitations are in the length of the experiments, which is 6 months, and the use of a constant L/S ratio. The average residence time of liquid water stored in the unfrozen base layer of rock glaciers can actually range from 5 to 13 months,^20^ and it is therefore to be expected that a more extended leaching time will result in greater release of solutes. As regards the L/S ratio, leachate concentrations of highly soluble species which have been removed from the solid are generally inversely proportional to the L/S ratio, whereas the final concentrations of species with low solubility are independent of the L/S ratio.^95^ The activity of chemolithotrophic bacteria on the catchment rock can also contribute to the increase in the soluble elements in water. Previous leaching experiments with rock sampled in the study catchment have shown that leaching efficiency is higher by 7–13% for Ca, 2–6% for Mg, and 5–6% for Mn in the presence of indigenous nitrifying or sulfur-oxidizing cultures relative to sterile conditions.^39^ Finally, a certain increase in ion concentration in the groundwater of complex shallow aquifer systems such as rock glaciers during wintertime may be result of the cryoconcentration phenomenon caused by a slowly advancing freezing front, leading to the progressive concentration of the residual solution.^96^ Overall, these and other related issues await further research to improve our knowledge of permafrost-related NARD in alpine environments.

## 4 Conclusions

This study suggests the critical role of prolonged water–rock interaction and cyclic freezing conditions, namely, diurnal FTCs, in the generation of acidic groundwater with toxic concentrations of heavy metals (e.g., Mn, Ni) by the rock glaciers in the crystalline catchment. In ExpFTC, repeated diurnal FTCs shortened the time of interaction of rock with liquid water by ∼2 times (to 3 months) compared with ExpT1 without FTCs (6 months). However, the leachate pH in ExpFTC was even lower (5.1) than that in ExpT1 (5.5) at the end of the experiments. Concentrations of Mn and Ni in the resultant leachates of ExpFTC were 1.4 and 1.8 times, respectively, higher than those of ExpT1. All of this suggests that the shorter time of interaction of rock with liquid water in ExpFTC was offset by the increased fracture-induced exposure of fresh rock surfaces, leading to enhanced sulfide dissolution and decreasing pH. The pH values and concentrations of Mn and Ni obtained in the ExpFTC and ExpT1 leachates were comparable to their seasonal variation in water of the discharge lake receiving groundwater from the rock glacier. Overall, this study confirms a geogenic origin of the toxic Mn and Ni concentrations in the discharge lake. It is the processes within the complex hydrological system of the rock glacier that are responsible for the huge content of these metals in lake water.

## Supporting Information Available

Concentrations of major ions and TDS in the resultant leachates and pH changes during the experiments (PDF)
