# Supplementary material for: Water–Rock Interaction and Freeze–Thaw Cycles as Drivers of Acid Rock Drainage Generation by a Rock Glacier in the European Alps
Source: ACS ES T Water. 2024 Nov 13;4(12):5264–74. doi: 10.1021/acsestwater.4c00263 (PMC11650631; doi:10.1021/acsestwater.4c00263)
Supplement: Supplementary file 1 — ew4c00263_si_001.pdf [file ew4c00263_si_001.pdf]

Supporting Information for:

Water–rock interaction and freeze–thaw cycles as drivers of acid rock drainage generation by a rock glacier in the European Alps

*Boris Ilyashuk** and *Elena Ilyashuk*

Department of Ecology, University of Innsbruck, Technikerstraße 25, 6020 Innsbruck, Austria

*Corresponding author:
Email: boris.ilyashuk@uibk.ac.at

Table S1. Concentrations (mean ± SD, *n* = 3) of major ions and total dissolved solids (TDS) in the resultant leachates. ExpFTC – under the action of diurnal freeze–thaw cycles, ExpT1 – at temperature of 1 ºC, and ExpT20 – at temperature of 20 ºC.

	Experiment		
	ExpFTC	ExpT1	ExpT20
Ca^{2+}, μeq L^{-1}	126 ± 32	301 ± 95	234 ± 92
Mg^{2+}, μeq L^{-1}	72 ± 16	140 ± 50	141 ± 50
Na^{+}, μeq L^{-1}	243 ± 25	166 ± 7	304 ± 59
K^{+}, μeq L^{-1}	39 ± 4	47 ± 13	92 ± 16
HCO_3^-, μeq L^{-1}	160 ± 5	370 ± 125	68 ± 37
Cl^-, μeq L^{-1}	216 ± 72	286 ± 69	269 ± 91
SO_4^{2-}, μeq L^{-1}	123 ± 10	68 ± 26	245 ± 84
TDS, mg L^{-1}	42 ± 7	56 ± 8	75 ± 17

Table S2. Changes in pH (mean ± SD, *n* = 3) over time during the leaching experiments: ExpFTC – under the action of diurnal freeze–thaw cycles, ExpT1 – at temperature of 1 ºC, and ExpT20 – at temperature of 20 ºC.

Time, days	Experiment		
	ExpFTC	ExpT1	ExpT20
0	6.87 ± 0.01	6.87 ± 0.01	6.87 ± 0.01
1	6.57 ± 0.02	6.38 ± 0.05	6.22 ± 0.04
3	6.21 ± 0.02	6.03 ± 0.03	5.74 ± 0.02
5	6.09 ± 0.02	5.94 ± 0.03	5.62 ± 0.02
7	5.97 ± 0.06	5.83 ± 0.04	5.44 ± 0.03
9	5.93 ± 0.09	5.89 ± 0.03	5.42 ± 0.09
11	5.88 ± 0.06	5.87 ± 0.04	5.48 ± 0.08
13	5.83 ± 0.05	5.80 ± 0.03	6.03 ± 0.04
15	5.65 ± 0.05	5.76 ± 0.02	6.20 ± 0.05
29	5.72 ± 0.05	5.67 ± 0.04	6.24 ± 0.06
43	5.69 ± 0.02	5.64 ± 0.04	6.21 ± 0.05
57	5.52 ± 0.05	5.52 ± 0.04	6.05 ± 0.09
71	5.49 ± 0.02	5.42 ± 0.03	5.95 ± 0.32
85	5.35 ± 0.08	5.34 ± 0.07	5.37 ± 0.17
99	5.28 ± 0.05	5.17 ± 0.07	4.98 ± 0.16
113	5.15 ± 0.07	5.31 ± 0.24	4.74 ± 0.05
127	5.13 ± 0.06	5.33 ± 0.21	4.72 ± 0.06
141	5.11 ± 0.05	5.34 ± 0.19	4.70 ± 0.08
155	5.04 ± 0.06	5.40 ± 0.22	4.70 ± 0.08
169	5.13± 0.06	5.46 ± 0.19	4.65 ± 0.06
183	5.11 ± 0.03	5.53 ± 0.17	4.51 ± 0.15
